# Can We Rely on Prophylactic Two-Level Vertebral Cement Augmentation in Long-Segment Adult Spinal Deformity Surgery to Reduce the Incidence of Proximal Junctional Complications?

**DOI:** 10.3390/medicina60060860

**Published:** 2024-05-24

**Authors:** Yoichi Tani, Nobuhiro Naka, Naoto Ono, Koki Kawashima, Masaaki Paku, Masayuki Ishihara, Takashi Adachi, Muneharu Ando, Shinichirou Taniguchi, Takanori Saito

**Affiliations:** Department of Orthopaedic Surgery, Kansai Medical University, 2-5-1 Shinmachi, Hirakata-City 573-1010, Japan; taniyoic@gmail.com (Y.T.); 3308sas@gmail.com (N.N.); naoto_ono0416@outlook.jp (N.O.); chelseajoesheva@gmail.com (K.K.); kmu.orthopaedics.pak@gmail.com (M.P.); ishihara0714@yahoo.co.jp (M.I.); adachita@hirakata.kmu.ac.jp (T.A.); mando@gaia.eonet.ne.jp (M.A.); tanigutskmsku@gmail.com (S.T.)

**Keywords:** adult spinal deformity (ASD), long-segment fusion, minimally invasive spine surgery (MIS), balloon kyphoplasty (BKP), prophylactic PMMA cement augmentation, proximal junctional kyphosis, proximal junctional failure, percutaneous pedicle screw, lateral lumbar interbody fusion

## Abstract

*Background and Objectives*: Proximal junctional kyphosis (PJK) and failure (PJF), the most prevalent complications following long-segment thoracolumbar fusions for adult spinal deformity (ASD), remain lacking in defined preventive measures. We studied whether one of the previously reported strategies with successful results—a prophylactic augmentation of the uppermost instrumented vertebra (UIV) and supra-adjacent vertebra to the UIV (UIV + 1) with polymethylmethacrylate (PMMA)—could also serve as a preventive measure of PJK/PJF in minimally invasive surgery (MIS). *Materials and Methods*: The study included 29 ASD patients who underwent a combination of minimally invasive lateral lumbar interbody fusion (MIS-LLIF) at L1-2 through L4-5, all-pedicle-screw instrumentation from the lower thoracic spine to the sacrum, S2-alar-iliac fixation, and two-level balloon-assisted PMMA vertebroplasty at the UIV and UIV + 1. *Results*: With a minimum 3-year follow-up, non-PJK/PJF group accounted for fifteen patients (52%), PJK for eight patients (28%), and PJF requiring surgical revision for six patients (21%). We had a total of seven patients with proximal junctional fracture, even though no patients showed implant/bone interface failure with screw pullout, probably through the effect of PMMA. In contrast to the PJK cohort, six PJF patients all had varying degrees of neurologic deficits from modified Frankel grade C to D3, which recovered to grades D3 and to grade D2 in three patients each, after a revision operation of proximal extension of instrumented fusion with or without neural decompression. None of the possible demographic and radiologic risk factors showed statistical differences between the non-PJK/PJF, PJK, and PJF groups. *Conclusions*: Compared with the traditional open surgical approach used in the previous studies with a positive result for the prophylactic two-level cement augmentation, the MIS procedures with substantial benefits to patients in terms of less access-related morbidity and less blood loss also provide a greater segmental stability, which, however, may have a negative effect on the development of PJK/PJF.

## 1. Introduction

As evidenced by recent studies [1,2], a minimally invasive surgery (MIS) triad consisting of anterior column realignment (ACR), lateral lumbar interbody fusion (LLIF), and percutaneous pedicle screw (PPS) fixation in a select group of adult spinal deformity (ASD) patients helped shorten the fusion length without compromising clinical and radiographic outcomes over a minimum 2-year follow-up period. However, another group of ASD patients required long-segment instrumented fusions to achieve balanced spinal alignment in both coronal and sagittal planes.

Glattes et al. (2005) [3] published the first major study to assess proximal junctional kyphosis (PJK) in ASD patients after long-segment thoracolumbar instrumented fusions, although earlier studies had focused on the analogous phenomenon in adolescents [4,5,6]. PJK and its potentially serious clinical sequelae, called proximal junctional failure (PJF), have since gained increasing attention as one of the most prevalent complications following ASD surgery.

Previous studies categorized acute PJK/PJF as either soft tissue failure caused by disruption of the disc and posterior ligamentous complex or hard tissue failure resulting from osseous fracture. The former typically occurred in constructs that terminated in the upper thoracic region, and the latter more commonly occurred in those that ended in the lower thoracic region [7,8,9,10,11]. If so, attempts to increase the mechanical strength of the vertebral bodies and the bone–screw connection would prevent PJK in constructs terminating at the lower thoracic spines. In fact, according to several clinical reports [11,12,13,14,15,16,17] as well as a biomechanical cadaveric study [18], two-level augmentation of the uppermost instrumented vertebra (UIV) and supra-adjacent vertebra to the UIV (UIV + 1) with polymethylmethacrylate (PMMA) cement may benefit patients by reducing the incidence of PJF. Based on these positive results, with high expectations, we combined the prophylactic two-level augmentation with MIS procedures in long-segment instrumented fusion for ASD, although it is not reported in the literature. The current report studied whether this combination worked well in 33 consecutive ASD patients who required long-segment fusion from the lower thoracic spine to the sacrum. Of these, the current outcome study included 29 patients who had a minimum 3-year postoperative follow-up evaluation.

## 2. Materials and Methods

### 2.1. Patients

This IRB-approved study included a series of 29 ASD patients (4 men and 25 women) who underwent a combination of lateral lumbar interbody fusion (LLIF), long-segment pedicle screw (PS) instrumentation from the lower thoracic spine to the pelvis, and two-level balloon-assisted vertebroplasty using PMMA cement with a minimum 3-year postoperative follow-up. They had the procedures either as a single-stage operation (9) or as a two-stage anterior (LLIF)-posterior operation one week apart (20) at a mean age of 72.6 (range 57–81) years from December 2015 to December 2016 at our institution (Table 1). The surgical teams with one of the three experienced spine surgeons performed the procedure.

We reviewed the medical records to gather information about clinical assessments. Elderly women with medical comorbidities and a low bone mineral density (BMD) at the hip accounted for the majority of the patients, reflecting our rapidly aging society. A total of 18 patients had 1–3 medical comorbidities, such as hypertension (13), coronary heart disease (7), hyperlipidemia (6), diabetes (3), rheumatoid arthritis (2), arteriosclerosis obliterans (1), autoimmune hepatitis (1), and colorectal cancer (1) (Table 1). The bone quality assessment also included the measurements of the Hounsfield units (HUs) on preoperative CT scans at the UIV, UIV + 1, and UIV + 2 [19,20,21]. The fusion length reached either 8 or 9 segments extending from the UIV either at T9 in 1 patient or T10 in 28 patients to the pelvis with S2-alar-iliac fixation in all patients (Table 1). Presurgical symptoms included low-back pain in all 29 patients, neurogenic claudication in 4 patients, and radicular buttock/thigh/leg pain and numbness in 15 patients. In 1 patient, a distinct weakness involved the psoas, quadriceps femoris, and tibialis anterior muscles unilaterally. Another 2 patients showed unilateral footdrop. A sensory examination for light touch and/or pinprick revealed the impairment over the L3, L4, and L5 dermatome unilaterally in 1, 4, and 8 patients, respectively.

### 2.2. Spine Imaging Studies

All patients underwent coronal and sagittal full-length digital radiographic studies in the free-standing clavicle position at least at 5 different time points: (1) preoperatively, (2) immediately (within 2 weeks) following the operation, (3) 1 year postoperatively, (4) 2 years postoperatively, and (5) 3 years postoperatively. Measurements consisted of coronal Cobb angles and sagittal spinopelvic parameters, including the pelvic incidence (PI), lumbar lordosis (LL) measured between L1 and S1, pelvic tilt (PT), thoracic kyphosis (TK) formed between T5 and T12, sagittal vertical axis (SVA), and the proximal junctional angle (PJA) measured between the caudal endplate of the UIV and cephalad endplate of the UIV + 2, as described by Glattes et al. [3]. In agreement with Martin et al. [13], we defined PJK as a kyphotic PJA ≥ 10° and a PJA increase by ≥ 10° at a follow-up evaluation compared with the immediately postoperative baseline measurement, but not with the preoperative baseline used originally by Glattes et al. [3] and in most of the subsequent reports. As described by Martin et al., surgical restoration of lumbar lordosis likely causes a reciprocal change in the lower thoracic spine, thereby overestimating the kyphotic PJA change if compared with the preoperative baseline. We defined PJF as the complications requiring surgical revision around the construct’s proximal level, as defined in most previous studies [14,16,17,22,23,24], although some studies categorized other symptomatic PJK as PJF regardless of whether the patients had surgical revision [7,25,26]. Radiographic assessments also included a lordotic “UIV angle” measured from the inferior endplate of the UIV to the horizontal on the early postoperative radiograph to investigate if a high UIV angle correlated with PJK/PJF [27].

We used Vue PACS version 11.4.1.1102 software (Carestream Health Inc., New York, NY, USA) to obtain these measurements.

### 2.3. Surgical Techniques

1. Anterior procedures (LLIF)

We used previously described techniques for LLIF at L1-2 through L4-5. The patient was placed in the true lateral position facing the side with a more caudal iliac crest [28,29]. With a 5 cm oblique single incision in line with the fibers of the external abdominal oblique muscle along the pen mark on the skin located at the center of the levels to be addressed, we performed a blunt dissection of the 3 abdominal muscle layers followed by developing the retroperitoneal space to identify the psoas muscle. We utilized an additional 3 cm incision over the L1–L2 disc space if necessary. The dilators were then introduced sequentially onto the disc space either through the psoas muscle (transpsoas approach) in 25 patients or anterior to the muscle (prepsoas approach) in 4 patients. We then opened the retractor blades, which were centered over the anterior two-thirds of the disc space as a table-mounted retractor under lateral fluoroscopic guidance. When using the transpsoas approach, an evoked potential analyzing system (NVM5 system; NuVasive, Inc., San Diego, CA, USA) was connected to each dilator, and threshold-based electromyographic monitoring of the lower-limb muscle was used to confirm safe passage of the dilators through the psoas muscle [1,2,29,30]. A thorough discectomy, performed carefully so as not to violate the subchondral bone of the endplates [1,2,29,31], included release of the contralateral anulus using a Cobb elevator and placing the widest possible implant that spanned the lateral margins of the apophyseal ring bilaterally to maximize endplate support [1,2,29,32,33,34]. We used porous titanium LLIF cages (CoRoent XL Titanium, NuVasive, Inc.) [35] with a 10° lordotic angle for 25 patients who underwent the transpsoas LLIF surgery and polyether ether ketone (PEEK) cages (Clydesdale; Medtronic Inc., Minneapolis, MN, USA) with a 6° lordotic angle for 4 patients who underwent LLIF with the prepsoas approach [36]. In both cases, the cage was packed with porous hydroxyapatite/collagen composite (Refit^®^; HOYA Technosurgical Co., Tokyo, Japan) [37,38] after soaking it in autogenous iliac bone marrow aspirate.

2. Posterior procedures, including prophylactic vertebral cement augmentation, pedicle screw placement, and S2-alar-iliac screw insertion

The patients subsequently underwent a posterior operation in the prone position either on the same day or about one week later. The procedures consisted of bilateral percutaneous PS (PPS) placements at all lumbosacral spines (L1 through S1) with S2-alar-iliac screw insertion and open vertebral PS placements and fusion at the lower thoracic spines (from T9 to T12 or from T10 to T12) with prophylactic two-level vertebral cement augmentation at the UIV and UIV + 1.

In the lumbosacral spines, the less imaging cannulated awl and probe (LICAP) system (Tanaka Corp., Tokyo, Japan) developed by us [39], coupled with nerve root monitoring with PPS stimulation [40,41], facilitated safe PPS placement without the use of fluoroscopy or computer-aided navigation systems. Meanwhile, we caudally extended the tiny skin incisions used for bilateral S1-PPS placements to identify the space between the neuroforamina of S1 and S2 as an insertion point, where fluoroscopic guidance allowed for optimal S2-alar-iliac screw placement [42].

In the thoracic spines, we used a standard midline approach for vertebral cement augmentation of the UIV and UIV + 1, bilateral PS insertions, and posterior fusion from UIV to L1 with autogenous morselized local bone harvested from the thoracic spinous processes. For two-level vertebral cement augmentation, we first placed a working cannula bilaterally through the pedicles of the UIV and UIV + 1 via a transpedicular route, followed by inserting a 15 mm balloon through the cannula. Inflating the balloon with radiopaque contrast under fluoroscopic control created a cavity within the osteoporotic vertebral body. We then introduced PMMA cement of an optimal viscosity into the void under imaging surveillance, watching for cement extravasation beyond the vertebral body margins [29,43,44,45]. In the UIV, we inserted a guidewire into the vertebral body through the cement introducer immediately after injecting the bone cement, before it hardened. Removing the cement introducer with the guidewire left behind, we inserted and tightened the cannulated PS of an appropriate diameter and length bilaterally over the guidewire. In the UIV + 1, we allowed the cement to completely set before removing the cement introducer.

### 2.4. Statistical Analysis

For statistical analyses, we used the chi-square test to analyze categorical data and the paired *t*-test and Mann-Whitney U-test to compare continuous data from the two groups, and repeated-measures ANOVA followed by the Tukey–Kramer honestly significant difference test to examine 3 or more groups of related data, with *p* < 0.05 considered significant. Means ± SEs and corresponding 95% CIs are reported.

## 3. Results

### 3.1. Changes in PJA

Of the twenty-nine patients, fifteen (52%) showed neither PJK nor PJF (non-PJK/PJF group), whereas eight patients (28%) met the PJK criteria (PJK group) and six (21%) required a revision operation for proximal junctional complications (PJF group) (Table 2). The non-PJK/PJF cohorts showed a significant increase in PJA postoperatively from the preoperative values (*p* = 0.0118), but did not indicate a further increase at 3 years postoperatively (*p* = 0.0883). In contrast, PJAs in the PJK and PJF groups showed no significant increases postoperatively (*p* = 0.1649 and 0.2465, respectively), but the angles resulted in subsequent increases at 3 years postoperatively in the PJK group and immediately before re-operation in the PJF group (both *p* < 0.0001) (Table 2). These results may indicate that an initial increase in PJK helps protect from a subsequent increase in PJA.

### 3.2. Changes in Sagittal Spinopelvic Parameters and Coronal Cobb Angles

As shown in Table 3, across all three groups, the patients preoperatively had lumbar hypolordosis (PI–LL mismatch > 20° [SRS-Schwab’s grade ++]), increased pelvic retroversion (PT > 25° [grade + or ++]), and impaired global sagittal balance (SVA > 95 mm [grade ++]) [46]. All the groups regained LL that was postoperatively associated with a reciprocal increase in TK and improvements of the three sagittal modifiers to grade 0 (i.e., PI–LL < 10°, PT < 20°, and SVA < 40 mm). The magnitude of sagittal modifier corrections seen at the early postoperative period tended to decline. Nevertheless, the improvements from the preoperative values still remained statistically significant for all three modifiers in the non-PJK/PJF group and for two modifiers, except for PT, in the PJK group at 3 years postoperatively. The PJF group also retained significantly better PI–LL and SVA until immediately before the re-operation. All the groups maintained the postoperative improvement of coronal Cobb angles <10° at the 3-year follow-up (non-PJK/PJF and PJK groups) or until immediately before the re-operation (PJF group).

### 3.3. PJK Group

PJK developed as a consequence of proximal disc failures in all eight PJK cohorts, with an additional vertebral collapse of UIV and UIV + 1 in one patient each. None of these radiographic PJK had accompanying neurologic dysfunction. They either remained clinically asymptomatic, even in one patient with vertebral collapse, or exhibited mild local pain indicating that the medicine had been effective (Table 4).

### 3.4. PJF Group

PJF affected the patients mostly in their 70s (average, 74 years; range, 69 to 78 years) and those with low mineral bone densities with a low T-score (average, –2.3; range, –3.8 to –1.4) (Table 5A). All PJF patients preoperatively had lumbar hypolordosis of PI–LL mismatch >10° and all but one had increased pelvic retroversion of PT > 20° and impaired global sagittal balance of SVA > 40 mm. The postoperative improvement reached coronal Cobb angle < 10° in all six patients, PI–LL < 10° in all but one, PT < 20° in all but two, and SVA < 40 mm in all but two (Table 5A), indicating that the PJFs do not seem to be attributable to the postoperative sagittal spinopelvic malalignment.

Four patients had segmental instability between UIV and UIV + 1 in combination with vertebral collapse of UIV, UIV + 1 or both. The remaining two patients developed PJF as a result of either vertebral collapse or segmental instability, but not both: case no. 1 showed only vertebral collapse of UIV and UIV + 2, and case no. 6 had segmental instability alone between UIV and UIV + 1. All the PJFs occurred within the 2-year postoperative period ranging from 2 to 20 months (average, 9.8 months) postoperatively, with no history of trauma preceding the change (Table 5B).

In contrast to the PJK cohort, all six PJF patients had a varying degree of neurologic deficits. Based on a functional grading of modified Franke grading system [47,48], one patient preserved motor function at a high grade (4+ to 5+) and normal voluntary bowel or bladder function (grade D3), two presented preserved motor function at the lowest grade (3+ of 5+) and/or with bowel or bladder paralysis with normal or reduced voluntary motor function (grade D1), and three presented preserved function with a less-than-fair motor grade (i.e., non-functional for any useful purpose) (grade C) (Table 5B). The revision operation for PJF consisted of proximal extension of instrumented fusion to T5 in five patients and to T3 in one patient, with neural decompression in four patients and without decompression in two patients. Half of them recovered to modified Frankel’s grade D3, and the other half to D2 with a minimum 3-year follow-up (Table 5B).

### 3.5. Analysis of Risk Factors for the Development of PJK and PJF

Table 6 summarizes the results of analyses of risk factors for PJK/PJF in our series of patients. They included (1) patient demographic factors, such as patients’ ages [48,49,50,51,52,53], BMD [48,50,54,55,56], and HUs [19,20,21]; and (2) radiographic factors, such as the postoperative UIV angle [27], the pre- and postoperative sagittal spinopelvic parameter values [48,54,57,58], and the differences between the two (i.e., risk of overcorrections) [3,48,54,57,59,60,61] for PJA, PI–LL, TK, and SVA. Consequently, none of the possible risk factors showed statistically significant differences between the non-PJK/PJF, PJK, and PJF groups (Table 6). The patients with the medical comorbidities listed in Table 1 were scattered across the three groups. Only one patient, who fell into the PJF group (case no. 5), had chronic oral steroid use of low-dose prednisone of 2.5 mg/day, which could exacerbate the osteoporosis, for autoimmune hepatitis.

### 3.6. Case Presentation

1. Case no. 6

**Figure 1:**
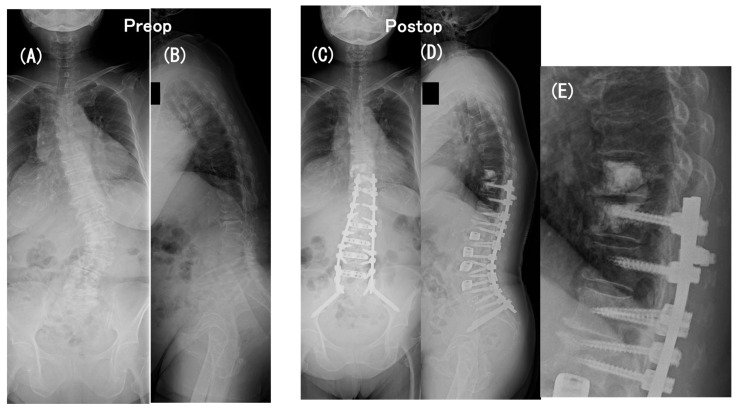
A 78-year-old woman with persistent low-back pain and loss of ambulatory endurance with scoliosis and severe sagittal spinopelvic imbalance. Coronal (**A**) and sagittal (**B**) full-length standing radiographs obtained preoperatively revealed the following: coronal Cobb angle = 42°, LL = 19°, PI = 56°, PI–LL = 37°, PT = 31°, TK = 18°, and SVA = 167 mm. Coronal (**C**) and sagittal (**D**) full-length standing radiographs and a close-up lateral radiograph (**E**) obtained at an early postoperative period. A two-stage operation, one week apart, comprised (1) the first-stage operation for the LLIF procedure at L1-2 through L4-5 with the patient in the lateral position and (2) the second-stage operation for posterior instrumentation with the patient placed prone, consisting of PPS fixation at L1 through S1, mini-open transforaminal lumbar interbody fusion (TLIF) at L5-S1 with S2-alar-iliac screw fixation, and a traditional open approach for PS fixation and fusion at T10 through T12 with prophylactic PMMA cement augmentation at T9 and T10. Postoperatively, the coronal Cobb angle decreased to 7° and sagittal spinopelvic parameters improved to 53° in LL, to 2° in PI–LL, to 16° in PT, to 46° in TK, and to 82 mm in SVA.

A 78-year-old woman with a low BMD (T-score, −1.4) had persistent low-back pain and loss of ambulatory endurance. Full-length standing radiographs revealed coronal as well as sagittal imbalance of the spine as follows: coronal Cobb angle = 42°, LL = 19°, PI = 56°, PI–LL = 37°, PT = 31°, TK = 18°, and SVA = 167 mm (Figure 1A,B).

She underwent a two-stage operation, one week apart: (1) the first-stage operation for the LLIF procedure was performed at L1-2 through L4-5 with the patient in the lateral position and (2) the second-stage operation for posterior instrumentation was performed with the patient placed prone, consisting of PPS fixation at L1 through S1, mini-open transforaminal lumbar interbody fusion (TLIF) at L5-S1 with S2-alar-iliac screw fixation, and a traditional open approach for PS fixation and fusion at T10 through T12 with prophylactic PMMA cement augmentation at T9 and T10. Postoperatively, the coronal Cobb angle decreased to 7° and sagittal spinopelvic parameters improved to 53° in LL, to 2° in PI–LL, to 16° in PT, to 46° in TK, and to 82 mm in SVA (Figure 1C–E).

**Figure 2:**
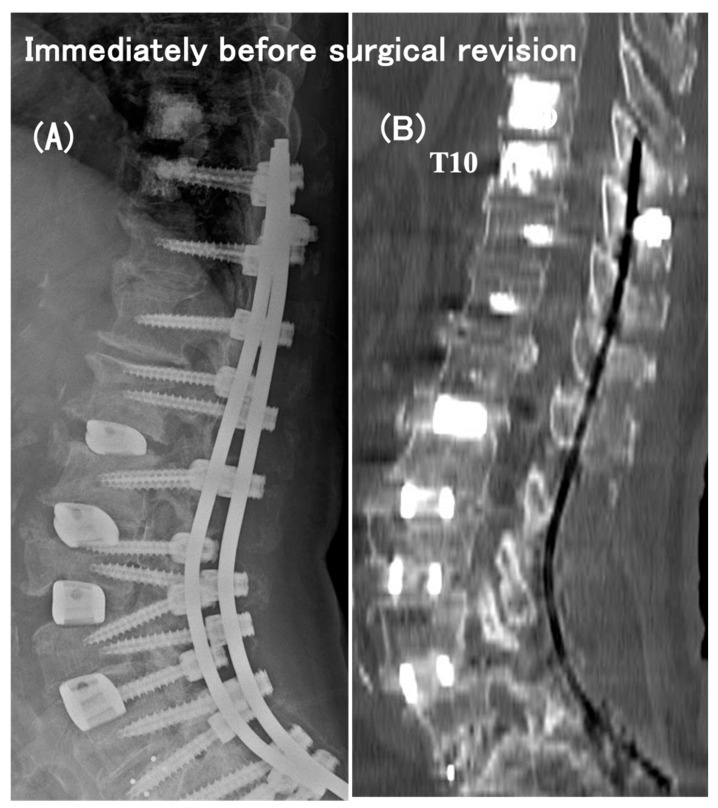
The plain radiograph (**A**) and a sagittal reformatted CT scan (**B**) obtained immediately before surgical revision for paraparesis of modified Frankel grade C, which developed as early as 2 months after the initial operation. These images indicated neither a PJK increase nor a vertebral fracture nor an implant failure.

**Figure 3:**
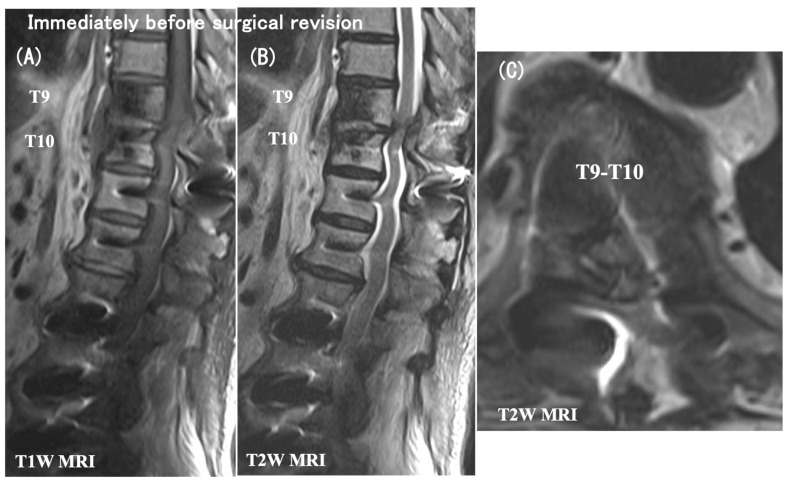
T1- (**A**) and T2-weighted (**B**) midsagittal MRI and a T2-weighted axial MRI at the T9–T10 intervertebral level (**C**) obtained immediately before surgical revision. These MRI scans showed distinct spinal cord compression at the T9–T10 intervertebral level (i.e., between UIV and UIV + 1).

**Figure 4:**
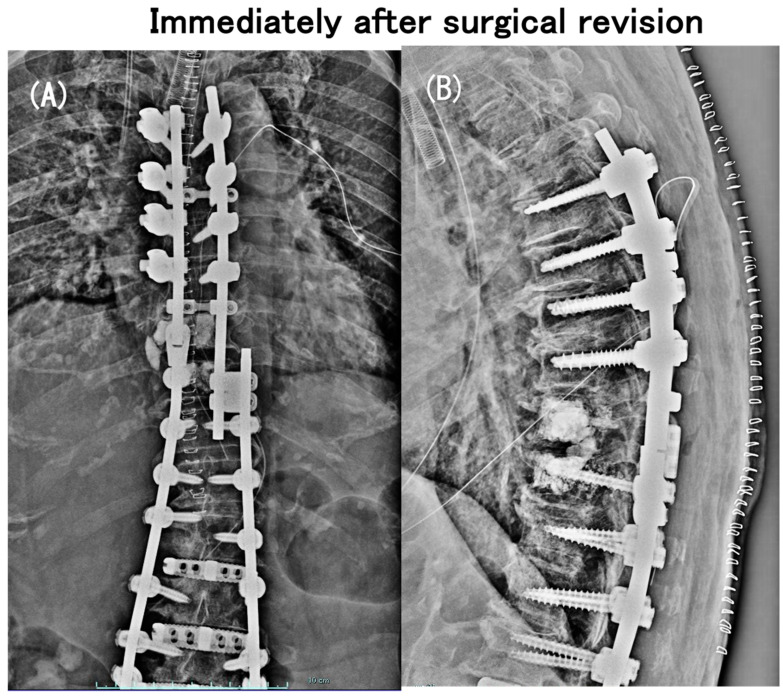
Coronal (**A**) and sagittal (**B**) full-length radiographs obtained at an early postoperative period of urgent surgical revision extending the posterior instrumented fusion proximally to T5 with spinal cord decompression.

**Figure 5:**
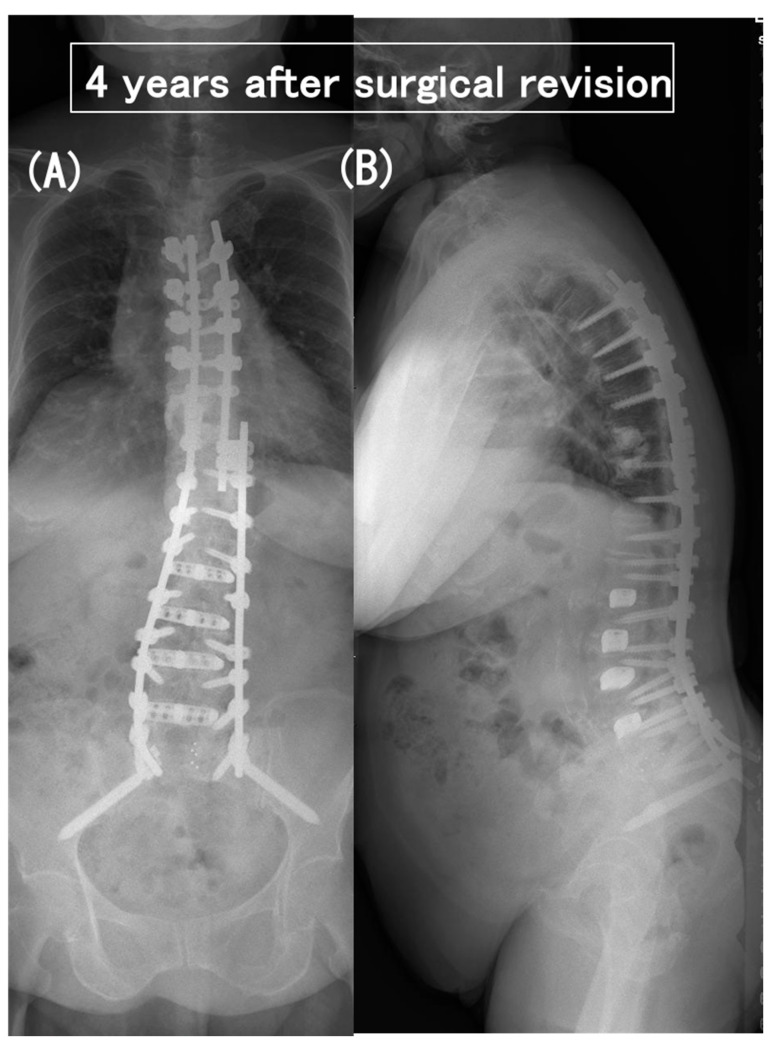
Coronal (**A**) and sagittal (**B**) full-length standing radiographs obtained 4 years after surgical revision. The paraparesis gradually improved from modified Frankel grade C to D2.

As early as 2 months postoperatively, despite the relief of preoperative low-back pain, she had a neurologic catastrophe of paraparesis with difficulty rising from the chair (modified Frankel grade C). The plain radiographs and CT scans indicated neither a PJA increase nor a vertebral fracture nor an implant failure (Figure 2), but the MRI scans showed a distinct spinal cord compression at the T9–T10 intervertebral level (i.e., between UIV and UIV + 1) (Figure 3). She needed to have emergency surgery, extending the posterior instrumented fusion proximally to T5 with spinal cord decompression (Figure 4). The paraparesis gradually resolved and she became ambulatory with a cane at the 4-year postoperative follow-up (modified Frankel grade D2) (Figure 5).

2. Case no. 4

**Figure 6:**
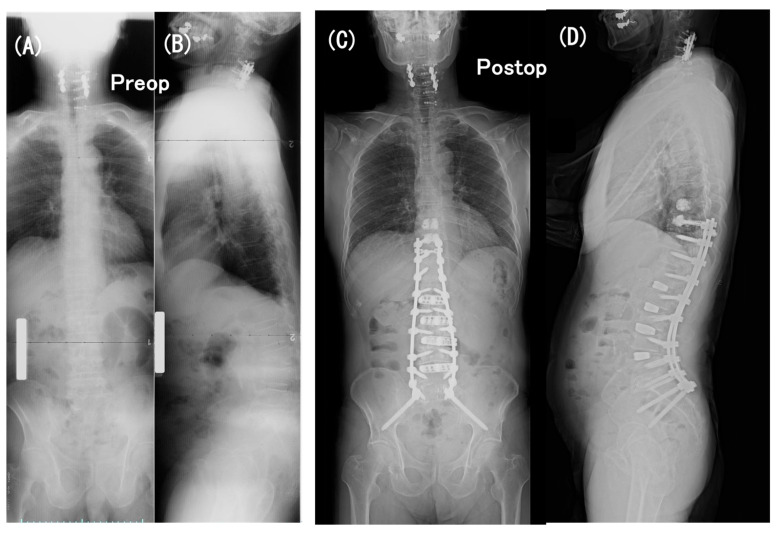
A 73-year-old man with persistent low-back pain and loss of ambulatory endurance with severe sagittal spinopelvic imbalance. Coronal (**A**) and sagittal (**B**) full-length standing radiographs obtained preoperatively revealed the following: LL = −19°, PI = 40°, PI–LL = 59°, PT = 38°, TK = 5°, and SVA = 118 mm. Coronal (**C**) and sagittal (**D**) full-length standing radiographs obtained at an early postoperative period. A two-stage operation, one week apart, comprised (1) the first-stage operation for LLIF procedure at L1-2 through L4-5 with the patient in the lateral position and (2) the second-stage operation for posterior instrumentation with the patient placed prone, consisting of PPS fixation at L1 through S1, mini-open transforaminal lumbar interbody fusion (TLIF) at L5-S1 with S2-alar-iliac screw fixation, and a traditional open approach for PS fixation and fusion at T10 through T12 with prophylactic PMMA cement augmentation at T9 and T10. Postoperatively, sagittal spinopelvic parameters improved to 47° in LL, to −6° in PI–LL, to 9° in PT, to 35° in TK, and to −8 mm in SVA.

A 73-year-old man with a low BMD (T-score, −3.1) had persistent low-back pain and loss of ambulatory endurance. Full-length standing radiographs revealed severe sagittal spinopelvic imbalance of the spine as follows: LL = −19°, PI = 40°, PI–LL = 59°, PT = 38°, TK = 5°, and SVA = 118 mm (Figure 6A,B).

**Figure 7:**
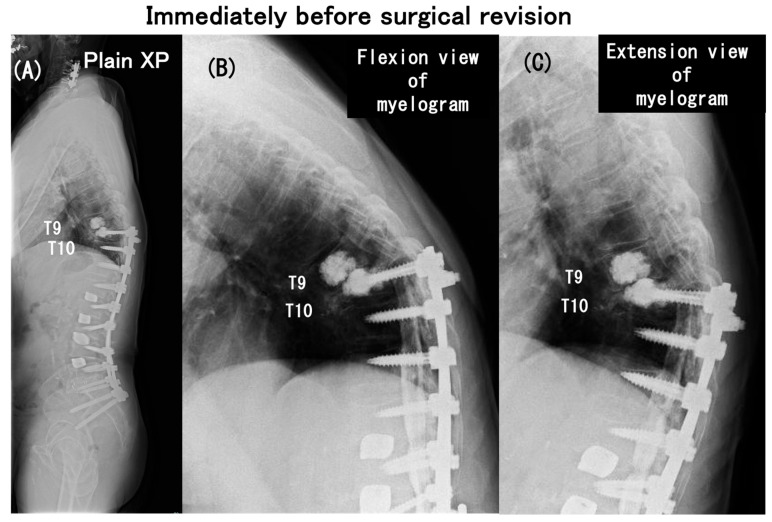
Plain radiograph (**A**) indicating PJK with segmental instability at the T9-T10 level (**A**) and lateral myelograms in flexion (**B**) and in extension (**C**) obtained immediately before surgical revision for paraparesis of modified Frankel grade D1, which developed as early as 6 months after the initial operation. The plain radiograph indicates PJK with segmental instability at the T9-T10 level (i.e., between UIV and UIV + 1) (**A**) and the lateral myelograms show a “block” to the flow of intrathecal contrast at the T9-T10 level with the T10 (i.e., UIV) fracture (**B**,**C**).

He underwent a two-stage operation, one week apart: (1) the first-stage operation for LLIF procedure at L1-2 through L4-5 was performed with the patient in the lateral position and (2) the second-stage operation for posterior instrumentation was performed with the patient placed prone, consisting of PPS fixation at L1 through S1, mini-open transforaminal lumbar interbody fusion (TLIF) at L5-S1 with S2-alar-iliac screw fixation, and a traditional open approach for PS fixation and fusion at T10 through T12 with prophylactic cement augmentation at T9 and T10 (Figure 7). Postoperatively, sagittal spinopelvic parameters improved to 47° in LL, to −6° in PI–LL, to 9° in PT, to 35° in TK, and to −8 mm in SVA (Figure 6C,D).

**Figure 8:**
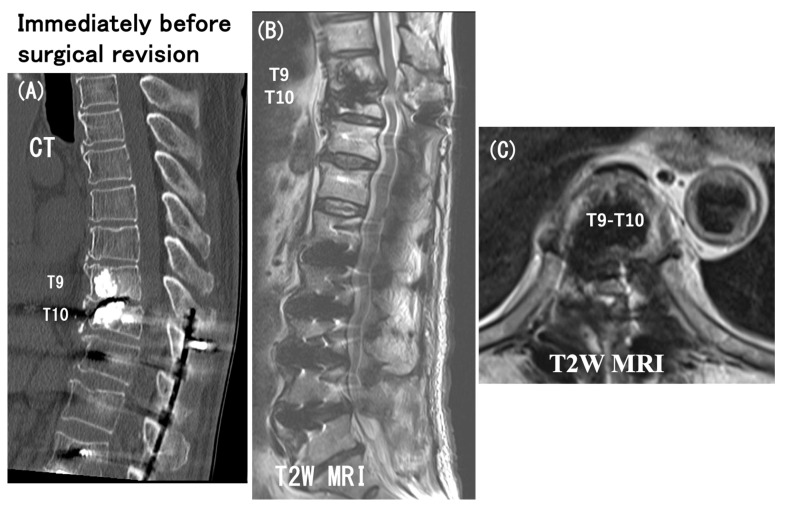
Sagittal reformatted CT scan (**A**), sagittal T2 weighted MRI (**B**), and axial T2 weighted MRI at T9-T10 level (**C**) obtained immediately before surgical revision. These images more clearly depict the UIV fracture without the screw pullout and distinct spinal cord compression at the T9-T10 intervertebral level.

**Figure 9:**
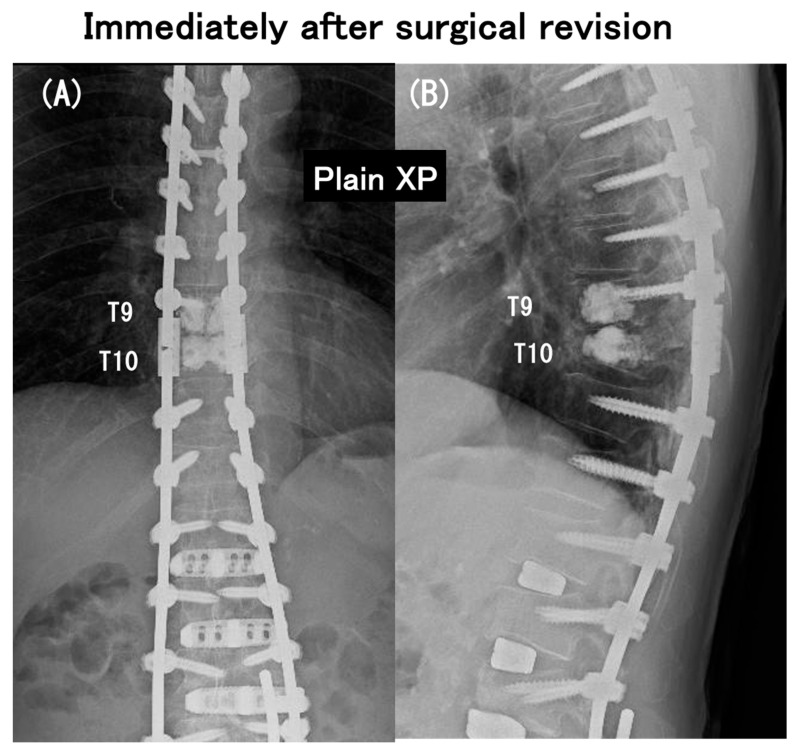
Coronal (**A**) and sagittal (**B**) radiographs obtained at an early postoperative period of urgent surgical revision extending the posterior instrumented fusion proximally to T5 with spinal cord decompression.

**Figure 10:**
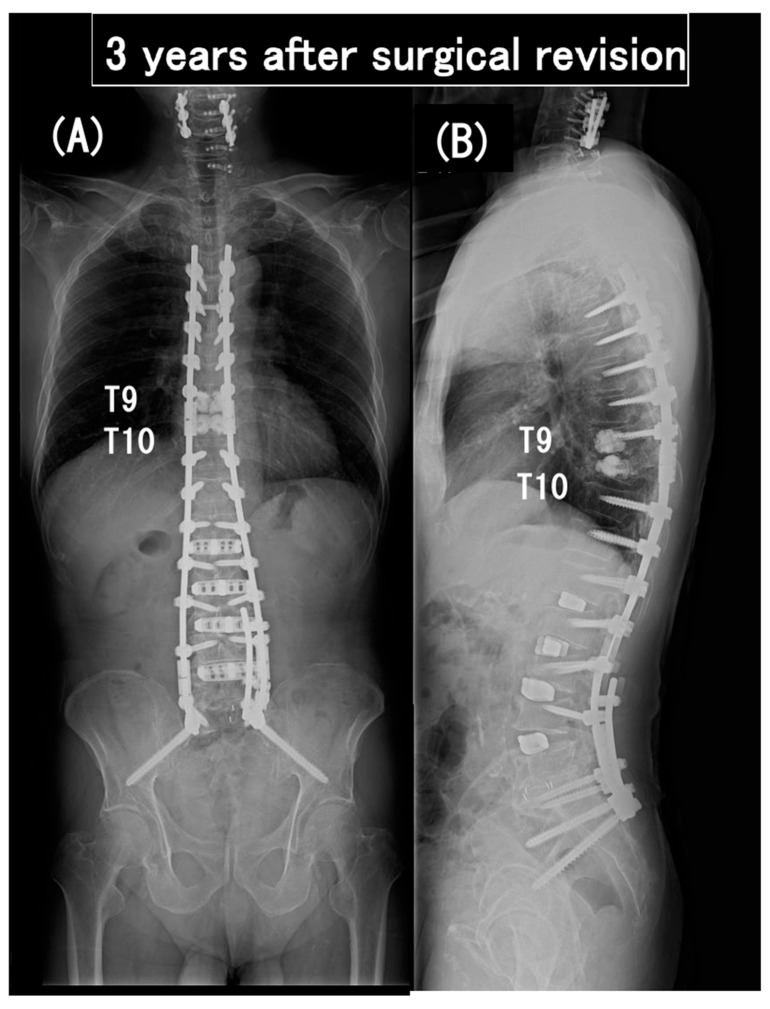
Coronal (**A**) and sagittal (**B**) full-length standing radiographs obtained 3 years after surgical revision. The paraparesis gradually improved from modified Frankel grade C to D2.

As early as 6 months postoperatively, despite the relief of preoperative low-back pain, he had spastic paraparesis (modified Frankel grade D1). The plain radiographs indicated PJK with segmental instability at the T9–T10 level (i.e., between UIV and UIV + 1) and lateral myelograms showed a “block” to the flow of intrathecal contrast at T9–T10 level with a T10 (UIV) fracture (Figure 7). The CT scan and T2 weighted MRIs in Figure 8 more clearly depicted the UIV fracture without the screw pullout and a distinct spinal cord compression at the T9–T10 intervertebral level. He needed to have emergency surgery, extending the posterior instrumented fusion proximally to T5 with spinal cord decompression (Figure 9). The paraparesis gradually resolved and he became ambulatory with a walking frame at the 3-year postoperative follow-up (modified Frankel grade D2) (Figure 10).

## 4. Discussion

Adjacent segment degeneration, also termed “transition syndrome”, represents a well-known complication after cervical or lumbar fusion surgery. Biomechanical studies demonstrated increased intradiscal pressures, angulation, and altered kinematics at the level adjacent to the prior fusion [53,62,63,64,65]. The thoracic vertebrae of T10 and above should have a greater resistance to adjacent segment failure than cervical and lumbar spines because of an additional mechanical support afforded through the true ribs, unlike floating ribs at T11 and T12 without costosternal articulation [66]. Despite this biomechanical advantage of the T9 and T10 vertebrae selected as the UIV in the current series, we encountered a surprisingly high total rate of PJK/PJF (48%) after long-segment instrumented fusion for ASD, even with 2-level prophylactic PMMA cement augmentation. One recent retrospective multicenter study [67] on a total of 641 ASD patients with long instrumentation extended to the pelvis (fusion length ≥ 9 levels) and a minimum 2-year follow-up reported the following outcomes: (1) 307 patients developed (47.9%) radiographic PJK (i.e., PJA > 10° and a PJA change > 10° with a preoperative baseline that differed from the definition used in the current study) at 2 years; and (2) 83 patients (12.9%) developed PJF defined as either severe radiographic PJK (i.e., PJA > 28° and a PJA change > 22°) at 2 years or clinical PJK (i.e., PJK that required revision surgery). Apart from PJK, for which many previous studies have failed to show that it leads to any statistical difference in health-related quality-of-life (HRQOL) outcomes compared with patients without PJK [54,57,68,69], such a high incidence (21%) of PJF in the current study presented a serious challenge to us. Since then, this disappointing result has made us avoid an excessive reliance on prophylactic vertebroplasty. The force of the long lever arm produced by the construct below extending from the lower thoracic spine to the pelvis probably must have exceeded the resistive capacity of the disc and posterior ligamentous complex and/or the augmented osteoporotic vertebral bodies at the proximal junction.

In contrast, some previous studies with traditional open surgery demonstrated that the same augmentation technique with PMMA served as an excellent means of PJK/PJF prevention. For example, as summarized in Table 7, PJK and PJF developed in only 8% and 5%, respectively, of the 38 patients with a minimum 2-year follow-up in study by Martin et al. [13], and 28% and 5% of the 39 patients with a minimum 5-year follow-up in the study by Raman et al. [15]. Moreover, in the comparative study by Theologis et al. [14], PJF with proximal junction fracture accounted for 0% of the 19 patients in the 2-level cement augmentation group vs. 22% of the 23 patients in the no-cement group (*p* = 0.02). Another retrospective cohort-matched surgical case series study by Ghobrial et al. [16] showed lower rates of PJK/PJF in the 38 patients in the PMMA group than in the 47 controls: 24% vs. 36% (*p* = 0.020) for PJK and 0% vs. 13% for PJF. Furthermore, as with our series of patients, all patients included in the study by Theologis et al. and most of those in the studies by Martin et al., Raman et al., and Ghobrial et al. underwent instrumented fusion extending to the pelvis (Table 7), which some reports identified as a biomechanical risk factor for PJK/PJF [49,52,54,57,68].

Why, then, did we have such a higher rate of PJF in our study? Sagittal spinopelvic alignment seems unlikely to be responsible for our results, because our patients postoperatively regained the ideal spinopelvic alignment values classified as “grade 0” [46] in all three crucial sagittal modifiers, showing PI–LL < 10°, PT < 20°, and SVA < 40 mm across all three groups (Table 3). None of the magnitudes of their surgical corrections significantly differed among the three groups (Table 6). An older population (mean, 72.6 years) with lower BMD (mean T-score, −1.6) in the current series than in the earlier studies may partly account for the worse outcomes (Table 1 and Table 7), although neither of them significantly differed among the three groups (Table 6). Posterior ligamentous complex disruption for harvesting the local bone graft from the thoracic spinous process may also have had an adverse effect [57,59,70,71].

In the above-referenced studies (Table 7), the prophylactic cement augmentation successfully achieved its primary objective of preventing the UIV and UIV + 1 from osseous fractures. In contrast, we had a total of seven patients with proximal junctional fracture, even though no patients showed implant/bone interface failure with pedicle screw pullout, probably through the effect of PMMA augmentation. This rigid screw–vertebral connection afforded by the PMMA at the UIV, however, may have been more harmful than screw pullout, endangering the adjacent segment integrity and causing its instability with a potentially catastrophic neurologic complication. Similarly, one study [72] documented a 70-year-old ASD woman who had all-pedicle-screw instrumentation at T10 through the pelvis with prophylactic PMMA augmentation at T9, T10, T11, L1, L5, and S1. She had a spastic paraparesis of modified Frankel grade C 30 days after hospital discharge as a result of T10 vertebral collapse and T9 vertebral subluxation without any inciting trauma. This case report, together with the outcomes of the current study, may indicate that spine surgeons should avoid excessively relying on the prophylactic effect of PMMA augmentation. No vertebral volume is ever 100% filled with the cement, leaving a weak trabecular bone mantle around the PMMA cement at risk of fracture [72,73].

A combined anterior–posterior (AP) fusion as opposed to a posterior-only approach constitutes a risk factor for the development of PJK/PJF, as identified in adults [49,68] as well as adolescents [74], although this remains controversial [52,54,57,67]. The risk may result from interbody fusion, which adds further rigidity anteriorly to the fusion constructs. From this viewpoint, one of the above-listed prior studies agreeable to PMMA augmentation included a total of 29 patients who had combined AP fusions at L4-5 and L5-S1, 25 in the control group, and 4 in the PMMA group [16]. The difference in the proportions of the patients with AP fusion showed statistical significance; 25 of 47 patients [53%] vs. 4 of 38 patients [11%]; *p* < 0.001. As a result, a significantly higher incidence was observed in the controls than in the PMMA group for both PJK (36% vs. 24%; *p* < 0.020) and PJF (13% vs. 0%; *p* < 0.031). Looking at their conclusion from another perspective, their data may have attested to the importance of a combined AP fusion as a risk factor for PJK/PJF rather than the benefit of PMMA augmentation as a measure for its prevention.

Unlike previous studies, our procedures incorporated interbody fusions with MIS-LLIF either with the transpsoas or prepsoas approach at L1-2 through L4-5 in all 29 patients. The interbody fusion in our LLIF procedure involved placement of the 10° lordotic angle titanium cage for the transpsoas LLIF or the 6° lordotic angle PEEK cage for the prepsoas LLIF [1,2,29]. We placed the tallest and widest possible implant that spanned the lateral margins of the apophyseal ring bilaterally [1,2,29]. This technique provides a greater segmental stability than a conventional anterior interbody fusion. This positive aspect of the LLIF procedure may conversely have a negative effect on the development of PJK/PJF.

The greater the construct rigidity, the more abrupt the change in stiffness from the instrumented to the uninstrumented mobile segments above. Previous biomechanical analyses indicate the importance of less rigid proximal fixation to allow for a more gradual transition to normal biomechanics, thereby reducing the incidence of PJK/PJF [10,70,75,76]. The PJK/PJF prevention strategies based on this notion include (1) the use of the transverse process hooks at the UIV instead of all-pedicle-screw instrumentation to accomplish a less rigid connection to the vertebra [60,69,75,77]; (2) the application of a transition rod that has a short taper to a smaller diameter at the rostral end to dampen the proximal transition forces from the UIV to the non-instrumented vertebrae above [70,75]; and (3) the addition of sublaminar tethers anchored at the UIV and above to reduce adjacent-segment loads [10].

In addition, our data in Table 2 suggested that a postoperative increase in PJA helped protect against a subsequent further increase in PJA, as seen in our non-PJK/PJF group. Consistent with this observation, previous studies reported the following two angles as a useful measure in predicting PJK/PJF: (1) The “UIV angle”, measured from the inferior endplate of the UIV to the horizontal, reported by Lewis et al. (2012) [27] tends to decrease with an increase in PJA. They revealed a strong association between smaller postoperative UIV angles and fewer UIV fractures. (2) The “rod contour angle (RCA)”, measured between UIV and L1, reported by Ishihara et al. (2021) [78] tends to increase with an increase in PJA. They revealed a significantly greater RCA in the non-PJK group than in the PJK group.

However, there remains a lack of a clearly-defined strategy for lowering the incidence of PJK and PJF. In fact, according to one recent review of a prospective multicenter database [67], the rate of radiographic PJK and/or PJF did not significantly decrease across the 10-year enrollment period of the ASD database. In the continuing controversy over various PJK/PJF prevention strategies, the current study suggests that PMMA cement augmentation needs caution when used in a combined AP fusion, in general, and in combination with MIS-LLIF, in particular. Only with such precaution will the selective use of PMMA cement augmentation play a meaningful role as a preventive measure against PJK/PJF.

## 5. Study Limitations

The current study has some limitations. First, a small sample size of only 29 ASD patients may make it difficult to generalize the results. Second, the current study lacks a control group without the prophylactic two-level vertebral cement augmentation for comparison. Third, our surgical strategy did not consistently follow the principle of MIS throughout the entire surgical course, involving a traditional open approach for PS fixation and fusion at T10 through T12 with harvesting the local bone graft from the thoracic spinous process. Therefore, the current study failed to elucidate how critical it is for PJK/PJF prevention to preserve the integrity of the posterior ligamentous complex at the proximal junction.

## 6. Conclusions

The current study analyzed whether the prophylactic two-level cement augmentation at UIV and UIV + 1 worked well when applied in conjunction with MIS-LLIF and all-pedicle-screw instrumentation from the lower thoracic spine to the pelvis. Compared with the traditional open surgical approach used in previous studies with a positive result for this additional prophylactic technique, the MIS procedures with substantial benefits to patients in terms of less access-related morbidity and less blood loss also provide a greater segmental stability, which, conversely, may have a negative effect on the development of PJK/PJF.

## Figures and Tables

**Table 1:** Patient demographics and operative details.

	29
Mean age, yrs (range)	72.6 (57–81)
Female sex, n (%)	25 (86.2)
Mean hip BMD, g/cm^2^ (range) ^§^	0.73 (0.42–1.1)
T-score	−1.6 (−3.8 ~ 1.6)
Mean HUs (±SE) ^ǂ^	
UIV	125.6 ± 7.5
UIV + 1	127.1 ± 7.4
UIV + 2	128.9 ± 7.6
Medical comorbidities, n	
Hypertension	13
Coronary heart disease	7
Hyperlipidemia	6
Diabetes	3
Rheumatoid arthritis	2
Arteriosclerosis obliterans	1
Autoimmune hepatitis	1
Colorectal cancer	1
No. of levels fused (motion segment)	8 or 9
UIV, n (%)	
T9	1 (0.4)
T10	28 (96.6)
LIV, n (%)	
S2 alar-iliac fixation	29 (100)
Anterior–posterior procedure, n (%)	
One-stage operation	9 (31.0)
Two-stage operation	20 (69.0)

^§^ Measured using dual-energy x-ray absorptiometry of the hip; ^ǂ^ measured on preoperative CT scans; HUs, Hounsfield units; UIV, uppermost instrumented vertebra; UIV + 1, supra-adjacent vertebra to the UIV; UIV + 2, the vertebra 2 level proximal to the UIV; LIV, lowest instrumented vertebra.

**Table 2:** Changes in the proximal junctional angle (PJA) for the non-PJK/PJF, PJK, and PJF groups.

	PJA ^§^, °			PJA ^§^, °		PJA ^§^, °	
	Preop	Early Postop	*p* Value	3 yrs Postop	*p* Value (Early vs. 3 yrs)	Immediately before Re-Operation	*p* Value (Early vs. Before Re-Operation)
Non-PJK/PJF group (n = 15)	3.9 ± 0.6	9.4 ± 1.4	0.0118 *	13.3	0.0883	NA	NA
PJK group (n = 8)	4.9 ± 1.0	10.6 ± 1.6	0.1649	28.1	<0.0001 *	NA	NA
PJF group (n = 6)	6.5 ± 1.2	11.7 ± 1.2	0.2465	NA	NA	37.6	<0.0001 *

^§^ The proximal junctional angle is defined as the sagittal Cobb angle measured between the caudal endplate of UIV and cephalad end plate of the vertebra 2 level proximal to the UIV (UIV + 2). * Statistically significant difference. ° degree; NA, not applicable.

**Table 3:** Preoperative to postoperative spinal alignment changes over a minimum 3-year follow-up period for the non-PJK/PJF and PJK groups or until immediately before the re-operation for the PJF group.

	Non-PJK/PJF Group (n = 15)				PJK Group (n = 8)				PJF Group (n = 6)		
	Preop	Postop			Preop	Postop			Preop	Postop	
Parameters		Early	1 yr	3 yrs		Early	1 yr	3 yrs		Early	Immediately before the Re-Operation
PI (°)	49.4 ± 3.4	48.1 ± 2.8	48.0 ± 3.0	48.9 ± 2.9	49.1 ± 3.9	47.5 ± 3.7	49.5 ± 3.2	50.0 ± 3.2	49.3 ± 2.3	49.6 ± 2.0	50.4 ± 1.9
LL (°)	5.3 ± 4.8	44.5 ± 2.6 **	43.0 ± 2.8 **	38.1 ± 2.4 **	16.1 ± 4.8	47.6 ± 2.4 **	46.4 ± 2.7 **	43.0 ± 2.5 **	7.5 ± 5.9	51.0 ± 4.3 **	47.6 ± 3.3 **
PI–LL (°)	44.1 ± 4.0	3.4 ± 2.2 **	5.1 ± 2.4 **	10.7 ± 2.7 **	32.7 ± 5.3	0.4 ± 4.5 **	3.1 ± 3.9 ^§§^	7.0 ± 4.2 **	41.9 ± 3.8	−1.4 ± 5.0 **	2.8 ± 3.3 **
PT (°)	35.5 ± 2.6	18.9 ± 2.5 **	21.9 ± 2.4 **	24.4 ± 1.8 **	25.8 ± 4.5	17.7 ± 3.0	20.6 ± 1.6	24.8 ± 2.6	30.3 ± 3.7	17.7 ± 3.2 ^†^	23.3 ± 2.3
TK (°)	16.9 ± 2.5	34.9 ± 2.7 **	37.2 ± 2.5 **	39.5 ± 2.4 **	18.1 ± 3.2	31.9 ± 3.8 ^†^	37.3 ± 3.1 ^§§^	50.3 ± 2.6 **	18.7 ± 4.7	41.4 ± 4.9 ^§^	46.0 ± 3.7 ^§^
SVA (mm)	110.2 ± 13.8	20.7 ± 6.1 **	39.1 ± 10.7 *	65.1 ± 16.9 ^†^	130.9 ± 20.0	37.1 ± 9.8 **	36.8 ± 7.2 **	83.9 ± 13.0 ^†^	105.6 ± 21.8	27.6 ± 13.4 ^††^	40.3 ± 12.6 ^††^
CA (°)	25.0 ± 4.9	5.0 ± 1.0 **	5.2 ± 1.0 **	7.0 ± 1.3 **	30.8 ± 7.5	4.2 ± 0.9 **	5.0 ± 1.3 **	5.2 ± 0.9 **	24.1 ± 8.5	5.8 ± 0.9 ^†^	4.7 ± 1.3 ^†^

Values are shown as the means ± standard errors (SEs). PI, pelvic incidence; LL, lumbar lordosis (between L1 and S1); PT, pelvic tilt; TK, thoracic kyphosis (between T5 and T12); SVA, sagittal vertical axis; CA, cobb angle; ** *p* < 0.0001 (versus preop); * *p* < 0.0005 (versus preop); ^§§^ *p* < 0.001(versus preop); ^§^ *p* < 0.005 (versus preop); ^††^ *p* < 0.01 (versus preop); ^†^ *p* < 0.05 (versus preop).

**Table 4:** Pathogenesis of PJK/PJF and the causes that required revision surgery for PJF.

		No. of Patients, n (%)	
		PJK (n = 8)	PJF (n = 6)
*Pathogenesis*	Bony failure (vertebral fracture)	2 (25)	5 (83)
	Proximal disc or ligament failure	8 (100)	5 (83)
	Implant/bone interface failure	0	0
*Disability for revision*	Neurologic compromise	0	5 (83)
	Severe pain	0	1 (17)

PJK, proximal junctional kyphosis.

**Table 5:** (**A**) Preoperative evaluations and early outcome data for the initial operation in 6 patients who developed PJF (PJF group). (**B**) Pathogenesis, revision operation, and the outcome for PJF developed in 6 patients (PJF group).

(A)																	
		Before the Initial Op								Initial Op		Early Postop					
Case No.	Age (yrs)/ Sex	BMD (g/cm^2^)/ T-Score	HU (UIV/UIV + 1/UIV + 2)	LL (°)	PI–LL (°)	PT (°)	TK (°)	SVA (mm)	CA (°)	UIV	1 or 2-Satge Op	LL (°)	PI–LL (°)	PT (°)	TK (°)	SVA (mm)	CA (°)
1	73/F	0.525/−3.8	100.7/104.4/114.3	2	43	38	22	157	55	T10	2	46	7	27	60	30	8
2	75/F	0.670/−2.2	95.6/104.0/110.0	13	39	35	13	100	15	T10	2	35	17	28	24	45	8
3	76/F	0.718/−1.8	116.1/108.9/135.4	20	31	25	15	63	25	T10	2	63	−14	14	43	14	3
4	73/M	0.423/−3.1	113.6/109.2/110.2	−19	59	38	5	118	4	T10	2	47	−6	9	35	−8	5
5	69/M	0.733/−1.6	106.3/113.9/123.5	10	42	15	39	29	4	T10	1	62	−14	13	41	3	3
6	78/F	0.728/−1.4	206.1/174.4/168.5	19	37	31	18	167	42	T10	2	53	2	16	46	82	7
Mean	74/NA	0.633/−2.3	123.0/119.1/127.0	7.5	41.8	30.3	18.7	105.7	24.2	NA	NA	51	−1.3	17.8	41.5	27.7	5.7
**(B)**																	
		**PJF**								**Re-Operation**							
**Case** **No.**		**Modified Frankel** **Grade ^§^**		**Vertebral** **Collapse**		**Segmental** **Instability**		**Time Interval** **Between the** **Initial Op and PJF** **(month)**		**Proximal** **Extension** **of Fusion**		**Decompression**		**Modified Frankel Grade**		**FU** **(months)**	
1		D3		T8, T10		–		13		T5		−		D3		79	
2		D1		T10		UIV–UIV + 1		20		T5		-		D3		37	
3		C		T9, T10		UIV–UIV + 1		4		T3		+		D2		77	
4		D1		T10		UIV–UIV + 1		6		T5		+		D2		40	
5		C		T9		UIV–UIV + 1		14		T5		+		D3		74	
6		C		–		UIV–UIV + 1		2		T5		+		D2		96	

PJF, proximal junctional failure; BMD, bone mineral density; HU, Hounsfield unit; PI, pelvic incidence; LL, lumbar lordosis (between L1 and S1); PT, pelvic tilt; TK, thoracic kyphosis (between T5 and T12); SVA, sagittal vertical axis; UIV, Uppermost instrumented vertebra; UIV + 1, supra-adjacent vertebra to the UIV; UIV + 2, the vertebra 2 levels proximal to the UIV; NA, not applicable; FU, follow-up; ^§^ modified Frankel grade [47]: A, complete motor and sensory loss; B, preserved sensation only, voluntary motor function absent; C, preserved motor less than fair grade (i.e., non-functional for any useful purpose); D1, preserved motor function at the lowest grade (3+ of 5+) and/or with bowel or bladder paralysis with normal or reduced voluntary motor function; D2, preserved motor function at midfunctional grade (3+ to 4+ of 5+) and/or neurogenic bowel or bladder dysfunction; D3, preserved motor function at a high grade (4+ to 5+) and normal voluntary bowel or bladder function; E, completely normal motor and sensory function (may still have abnormal reflexes).

**Table 6:** Analysis of risk factors for development of PJK and PJF.

	Group			*p* Value		
Poteatial Risk Factors	Non-PJK/PJF Group (n = 15)	PJK Group (n = 8)	PJF Group (n = 6)	Non-PJK/PJF vs. PJK	Non-PJK/PJF vs. PJF	PJK vs. PJF
Mean age, yrs (range)	72.7 (57–81)	71.5 (62–75)	74.0 (69–78)	0.8530	0.8712	0.6542
Mean hip BMD, g/cm^2^ (range) ^§^	0.74 (0.65–1.1)	0.78 (0.66–0.88)	0.63 (0.42–0.73)	0.8366	0.3858	0.2384
T-score	−1.5 (−3.6 ~ 1.6)	−1.3 (−0.5 ~ −2.4)	−2.3 (−3.8 ~ −1.4)	0.9156	0.2461	0.1912
Mean HUs (±SE) ^‡^						
UIV	127.4 ± 10.2	124 ± 14.5	123.1 ± 16.9	0.9789	0.9725	0.9990
UV + 1	126.5 ± 11.6	134.2 ± 12.6	119.1 ± 11.2	0.8964	0.9216	0.7617
UIV + 2	130.3 ± 12.1	127.7 ± 13.7	127 ± 9.2	0.9883	0.9847	0.9995
Mean PJA (±SE), °						
Preop	3.9 ± 0.6	4.9 ± 1.0	6.5 ± 1.2	0.6889	0.1159	0.4756
Early Postop	9.4 ± 1.4	10.6 ± 1.6	11.7 ± 1.2	0.8367	0.5722	0.8956
Difference	5.4 ± 1.5	5.7 ± 2.1	5.2 ± 1.6	0.9947	0.9941	0.9838
Mean PI–LL (±SE), °						
Preop	44.1 ± 4.0	32.7 ± 5.3	41.9 ± 3.8	0.1844	0.9425	0.4765
Early Postop	3.4 ± 2.2	0.4 ± 4.5	−1.4 ± 5.0	0.8017	0.6314	0.9488
Difference	−40.7 ± 4.7	−32.3 ± 6.2	−43.2 ± 6.3	0.5247	0.9536	0.4919
Mean TK (±SE), °						
Preop	16.9 ± 2.5	18.1 ± 3.2	18.7 ± 4.7	0.9626	0.9329	0.9940
Early Postop	34.9 ± 2.7	31.9 ± 3.8	41.4 ± 4.9	0.7909	0.4341	0.2427
Difference	18.0 ± 3.2	13.8 ± 4.8	22.8 ± 5.5	0.7399	0.7283	0.4155
Mean SVA (±SE), mm						
Preop	110.2 ± 13.8	130.9 ± 19.7	105.6 ± 21.8	0.6603	0.9831	0.6655
Early Postop	20.7 ± 6.1	37.1 ± 9.8	27.6 ± 13.4	0.3562	0.8552	0.7908
Difference	−82.9 ± 12.5	−93.8 ± 20.0	−78.0 ± 17.1	0.8699	0.9779	0.8273
Mean UIV angle (±SE) ^†^, °						
Early Postop	7.5 ± 1.9	10.5 ± 2.6	13.1 ± 3.0	0.6309	0.2690	0.7822

^§^ Measured using dual-energy x-ray absorptiometry of the hip; ^‡^ measured on preoperative CT; ^†^ measured from the inferior endplate of the UIV to the horizontal; BMD, bone mineral density; HUs, Hounsfield units; UIV, uppermost instrumented vertebra; UIV + 1, supra-adjacent vertebra to the UIV; UIV + 2, the vertebra 2 level proximal to the UIV; PI, pelvic incidence; LL, lumbar lordosis measured between L1 and S1; PJA, proximal junctional angle defined as the sagittal Cobb angle measured between the caudal endplate of UIV and cephalad end plate of UIV + 2; TK, thoracic kyphosis measured between T5 and T12; SVA, sagittal vertical axis; ° degree.

**Table 7:** A comparison of patient demographics, operative details, and outcomes between the current study and 4 previous reports with a positive result for prophylactic two-level cement augmentation at UIV and UIV + 1.

Parameters	Two Groups (2-Level Cement Group /Control Group)	Current Study	Previous Reports in Favor of Prophylatic Two-Level Vertebral Cement Augmentation			
			Martin CT, et al. (2013) [13]	Theologis AA. and Burch S. (2015) [14]	Raman T, et al. (2017) [15]	Ghobrial GM, et al. (2017) [16]
No of patients, n	2-level cement No cement	29 0	38 0	19 23	39 0	38 47
Surgical technique, (n)	NA	MIS-LLIF, PPS, PSF with PS at T10 –T12	PSF with PS (27) + ALIF (11)	PSF with PS (27) + 3-CO (15)	PSF with PS (28) + ALIF (11)	PSF with PS (56) + ALIF (29) Sublaminar hooks at UIV (21)
Age at operation, yrs [mean (range)]	2-level cement No cement	72.6 (57–81) NA	64.4 (41–80) NA	68.2 (55–79) 59.8 (33–77)	65.6 (41–87) NA	71.0 58.3
Follow-up, mos [min or mean (range)]	2-level cement No cement	>36 NA	32.3 (24–80) NA	14.8 (6.6–32.8) 24.9 (8.3–55.3)	>60 NA	24.2 27.9
No. of levels fused, n [mean (range or min)]	2-level cement No cement	(8–9) NA	(approx 8–9) NA	9 7.7 (5–10)	(approx 8–9) NA	9 (>5) 9 (>5)
Pelvic fixation, n (%)	2-level cement No cement	29 (100) NA	32 (84) NA	19 (100) 23 (100)	33 (85) NA	36 (95) 45 (96)
PJF, n (%)	2-level cement No cement	6 (21) NA	2 (5) NA	0 (0) 5 (22)	2 (5) NA	0 (0) 6 (13)

n, numbers; yrs, years; mos, months; min, minimum; PJF, proximal junctional failure; NA, not applicable; MIS, minimally invasive surgery; LLIF, lateral lumbar interbody fusion; PS, pedicle screw placement; PPS, percutaneous pedicle screw placement; PSF, open posterior spinal fusion; ALIF, open anterior lumbar interbody fusion; 3-CO, 3-column osteotomy; UIV, uppermost instrumented vertebra; UIV + 1, supra-adjacent vertebra to the UIV. NA, not applicable.

## Data Availability

The original contributions presented in the study are included in the article, further inquiries can be directed to the corresponding author.
